# Protective effect of antihypertensive drugs on the risk of Parkinson’s disease lacks causal evidence from mendelian randomization

**DOI:** 10.3389/fphar.2023.1107248

**Published:** 2023-02-23

**Authors:** Zheng Jiang, Xiao-Jing Gu, Wei-Ming Su, Qing-Qing Duan, Yan-Lin Ren, Ju-Rong Li, Li-Yi Chi, Yi Wang, Bei Cao, Yong-Ping Chen

**Affiliations:** ^1^ Department of Neurology, West China Hospital, Sichuan University, Chengdu, Sichuan, China; ^2^ Lab of Neurodegenerative Disorders, Institute of Inflammation and Immunology (III), Frontiers Science Center for Disease-Related Molecular Network, West China Hospital, Sichuan University, Chengdu, Sichuan, China; ^3^ Centre for Rare Diseases, West China Hospital, Sichuan University, Chengdu, Sichuan, China; ^4^ Mental Health Center, West China Hospital, Sichuan University, Chengdu, Sichuan, China; ^5^ Department of Pathophysiology, West China College of Basic medical sciences and Forensic Medicine, Sichuan University, Chengdu, China; ^6^ Department of Geriatrics, Dazhou Central Hospital, Dazhou, Sichuan, China; ^7^ Department of Neurology, Xijing Hospital, Air Force Military Medical University, Xi’an, Shanxi, China

**Keywords:** Parkinson’s disease, age at onset, Mendelian randomization, blood pressure, antihypertensive medications

## Abstract

**Background:** Evidence from observational studies concerning the causal role of blood pressure (BP) and antihypertensive medications (AHM) on Parkinson’s disease (PD) remains inconclusive. A two-sample Mendelian randomization (MR) study was performed to evaluate the unconfounded association of genetic proxies for BP and first-line AHMs with PD.

**Methods:** Instrumental variables (IV) from the genome-wide association study (GWAS) for BP traits were used to proxy systolic BP (SBP), diastolic BP, and pulse pressure. SBP-associated variants either located within encoding regions or associated with the expression of AHM targets were selected and then scaled to proxy therapeutic inhibition of angiotensin-converting enzyme inhibitors, angiotensin receptor blockers, β-blockers, calcium channel blockers, and thiazides. Positive control analyses on coronary heart disease (CHD) and stroke were conducted to validate the IV selection. Summary data from GWAS for PD risk and PD age at onset (AAO) were used as outcomes.

**Results:** In positive control analyses, genetically determined BP traits and AHMs closely mimicked the observed causal effect on CHD and stroke, confirming the validity of IV selection methodology. In primary analyses, although genetic proxies identified by “encoding region-based method” for β-blockers were suggestively associated with a delayed PD AAO (Beta: 0.115; 95% CI: 0.021, 0.208; *p* = 1.63E-2; per 10-mmHg lower), sensitivity analyses failed to support this association. Additionally, MR analyses found little evidence that genetically predicted BP traits, overall AHM, or other AHMs affected PD risk or AAO.

**Conclusion:** Our data suggest that BP and commonly prescribed AHMs may not have a prominent role in PD etiology.

## Introduction

Parkinson’s disease (PD) is one of the most prevalent neurodegenerative diseases lacking any neuroprotective treatments (Pringsheim et al., 2014), while hypertension ranks among the leading risk factors for all-cause death and disability-adjusted life-years worldwide (GBD 2017 Risk Factor Collaborators, 2018). Since the prevalence of PD and hypertension increases with age, their coexistence in the elderly is not uncommon. Therefore, understanding whether hypertension and antihypertensive medications (AHM) were causal for PD will make the medical decision more reasonable in clinical practice.

The role of hypertension and AHMs in PD has long been debated (Simon et al., 2007; Ton et al., 2007). Summary meta-analyses of epidemiologic studies indicated hypertension might increase the risk for PD (Hou et al., 2018; Chen et al., 2019). Meanwhile, some AHMs, such as calcium channel blockers (CCB), have emerged as prioritized repurposing options for PD prevention (Swart and Hurley, 2016; Katsi et al., 2021). However, considering the limited number of prospective cohort studies, these findings should be cautiously interpreted. Additionally, traditional observational studies are prone to residual confounding and reverse causation (Lawlor et al., 2008) and lack insights into the role of drug targets for specific AHMs.

Mendelian randomization (MR) is an analytical tool proposed to overcome some weaknesses associated with conventional observational studies on estimating the causal effects of risk factors (Davies et al., 2018). In this approach, genetic alleles are randomly assorted during meiosis and thus reducing bias resulting from conventional confounding factors or reverse causality. Meanwhile, analogous to a randomized controlled trial (RCT), the MR method has also been applied to develop a novel indication of the existing drugs by applying randomly assorted variants in the drug target gene (Storm et al., 2021).

Without preventive or disease-modifying interventions (Sardi et al., 2018), prevention strategies targeting modifiable risk factors and repurposing the existing drugs to novel indications are promising for PD. Hence, by using two-sample MR analyses, the aims of this study were to 1) investigate the direct causal link between blood pressure (BP) traits and PD risk and age at onset (AAO) and 2) examine the causal effect of different AHM classes on PD risk and AAO, all of which will benefit for PD in diagnosis, intervention, and prognostic assessment.

## Methods

### Data for exposure

#### Instrument selection for blood pressure

We extracted single nucleotide polymorphisms (SNP) of BP based on summary statistics in a genome-wide association study (GWAS) meta-analysis of 757,601 individuals from the International Consortium of Blood Pressure database and UK Biobank (Evangelou et al., 2018). In this study, BP traits incorporated systolic BP (SBP), diastolic BP (DBP), and pulse pressure (PP), which have been adjusted for AHM use by adding 15 and 10-mmHg to SBP and DBP, respectively. We restricted the set of SNPs to be significantly associated with the exposure with a *p*-value reaching genome-wide significance (*p* < 5 × 10^–8^), and the threshold of linkage disequilibrium (LD) was set at *R* ^*2*^ = 0.001 using the 1,000 Genomes European reference panel. In addition, the above study adjusted effect estimates for body mass index (BMI), potentially introducing collider bias as BMI is causal for both elevated BP and PD, so a sensitivity analysis was performed using alternative UK Biobank GWAS summary statistics of SBP (N = 436419) and DBP (N = 436424) not adjusted for BMI(Mitchell et al., 2019).

#### Instrument selection for antihypertensive medications

Based on recent work by (Gill et al., 2019) and (Walker et al., 2019), we further chose genetic variants as proxies for the SBP lowering effects of first-line drugs for hypertension (Wright et al., 2018), including angiotensin-converting enzyme inhibitors (ACEI), angiotensin receptor blockers (ARB), β-blockers (BB), CCB, and thiazide diuretic agents (Supplementary Table S1).

In the main analyses (Gill et al., 2019), encoding or regulatory regions (promoters and enhancers) of pharmacologically active targets for these drugs were identified through the DrugBank database (Wishart et al., 2018) (http://www.drugbank.ca/; DrugBank V5.1.9) (67 target genes in total, Supplementary Table S1) and the GeneCards online platform (Fishilevich et al., 2017) (https://www.genecards.org/; GenHancer V5.9) (Supplementary Table S2). For all the identified variants in each gene, only variants significantly associated with SBP (*p* < 5 × 10^–8^) and clumped to an LD threshold of *R* ^*2*^ < 0.4 were considered candidate proxies for each drug class (Burgess et al., 2018; Nowak and Arnlov, 2018; Georgakis et al., 2020). More stringent LD thresholds (*R* ^*2*^ < 0.1 and *R* ^*2*^ < 0.001, respectively) were conducted in sensitivity analyses.

In the additional analyses (Walker et al., 2019), *cis*-expression quantitative trait loci (eQTL) of AHM target genes identified above by DrugBank for each tissue were extracted from the latest GTEx dataset (GTEx Consortium et al., 2017) (https://www.gtexportal.org/home/datasets; release V8, dbGaP Accession phs000424.v8.p2) (67 target genes in 49 tissues, Supplementary Table S3). In the positive control and instrument validation step, we excluded SNPs with a null effect on SBP for each AHM target gene in two-sample MR analyses (*p* > 0.05). The selection strategy of LD thresholds was similar to the main analyses.

For all the selected instrumental variables (IV) in this study, *F*-statistics were above 10, indicating that weak instrumental bias is minimal (Bowden et al., 2016).

#### Positive control analysis

Positive control analyses were performed to validate the IV selection in our study. Firstly, for the IVs of blood traits and AHM targets identified by “encoding region-based method”, we examined the association of exposures of interest with coronary heart disease (CHD) and stroke because hypertension is an established risk factor for both CHD and stroke. In addition, for the IVs of AHM targets identified by “eQTL-based method,”, we examined the causative association of exposures of interest with SBP since the BP-lowering effect is the well-proven effect of AHMs.

### Data for outcome

For the main outcome of PD risk, we used the largest and most comprehensive summary statistics data from a meta-analysis GWAS performed by the International Parkinson’s Disease Genomics Consortium, including 33,674 PD cases and 449,056 controls (Nalls et al., 2019). In addition, previous studies indicate that the genetic risk of PD is correlated with PD AAO (Escott-Price et al., 2015; Smolders et al., 2021), so PD AAO was used as the secondary outcome from a GWAS meta-analysis of 28,568 cases (Blauwendraat et al., 2019).

For the positive control outcomes, GWAS summary data for CHD were based on the CARDIoGRAMplusC4D Consortium, which conducted a meta-analysis of 60,801 CHD cases and 123,504 controls (Nikpay et al., 2015). Summary data of stroke were drawn from a recent large-scale meta-analysis of GWAS (MEGASTROKE) confined to European populations of 40,585 cases and 406,111 controls (Malik et al., 2018).

### Primary analysis

**FIGURE 1:**
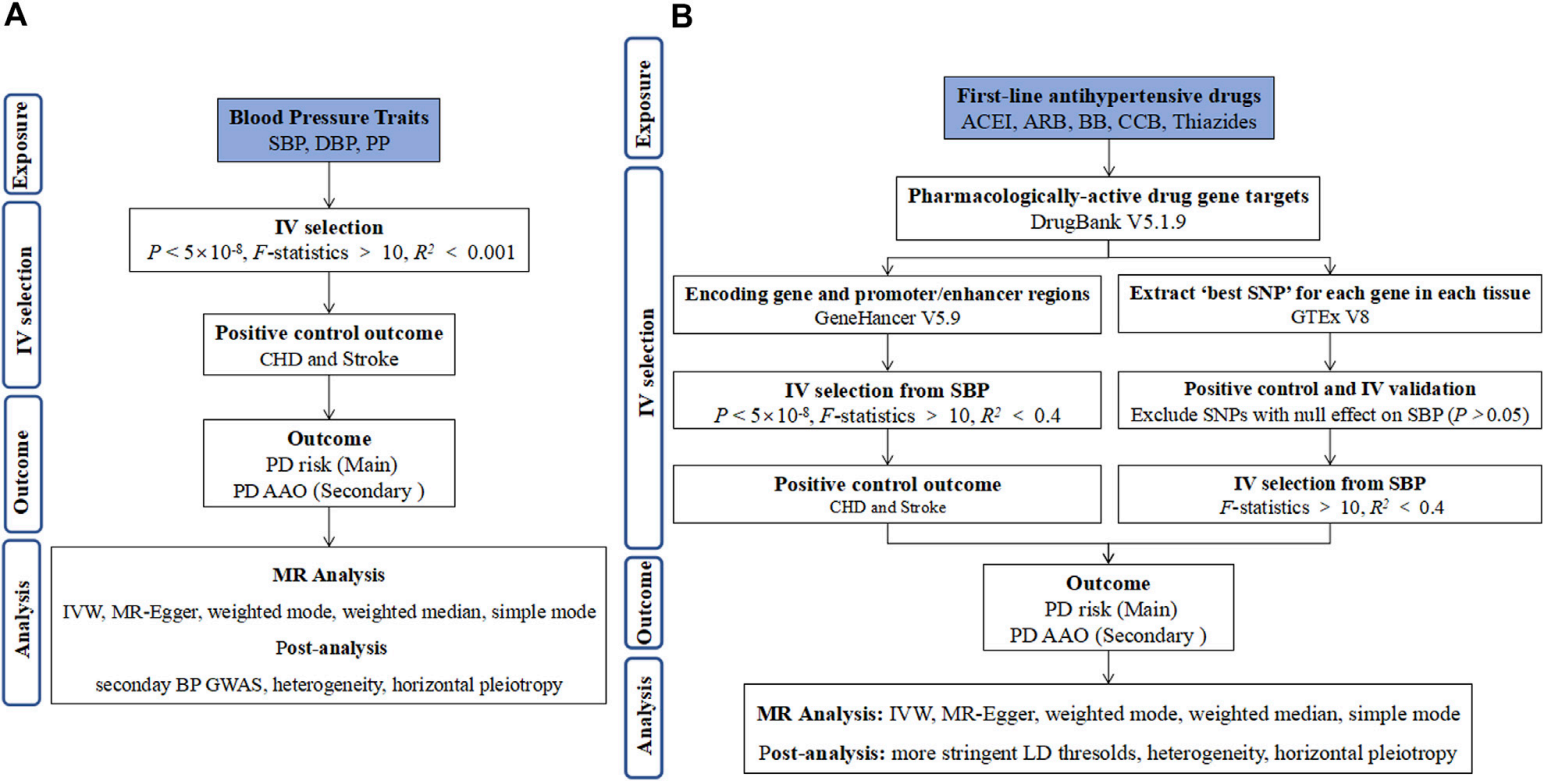
Flow diagram of the process for the two-sample MR analyses of blood pressure **(A)** and antihypertensives **(B)** with Parkinson’s disease. Abbreviations: IV, instrumental variable; SBP, systolic blood pressure; DBP, diastolic blood pressure; PP, pulse pressure; ACEI, angiotensin-converting enzyme inhibitors; ARB, angiotensin receptor blockers; BB, β-blockers; CCB, calcium channel blockers; CHD, coronary heart disease; MR, Mendelian randomization; IVW, inverse variance weighted; LD, linkage disequilibrium; PD, Parkinson’s disease; AAO, age at onset.

Two-sample MR analyses were performed in this study. Three assumptions were established, including that the genetic instruments were associated with the exposure of interest, were independent of potential confounders, and could only affect the outcome through the exposure of interest and not through other pathways. Firstly, we examined the causal effect of BP traits on PD (Figure 1A). Second, we examined the causal effect of overall AHM and different AHMs on PD (Figure 1B). The Wald ratio test was used to calculate the causative effect when a single IV was available, while the multiplicative random effects inverse variance weighted (IVW) method was performed as the main analysis when multiple IVs were available (Lee, 2020). IVW generalized the Wald ratio through a meta-analysis process, and it is the most efficient analysis method with valid IVs because it accounts for heterogeneity in the variant-specific causal estimates (Burgess et al., 2019; Lee, 2020).

### Sensitivity analysis

Sensitivity analyses accounting for certain violations of the MR assumptions due to different pleiotropy scenarios, including the MR-Egger regression, weighted median, simple mode, and weighted mode methods, were conducted to assess the robustness of the findings (Burgess et al., 2019). Furthermore, we used the MR Egger intercept and Cochran Q statistic to test the presence of directional pleiotropy and heterogeneity, respectively. If outlier IVs were detected using the MR pleiotropy residual sum and outlier (MR-PRESSO) test (Verbanck et al., 2018), the IVW MR analysis was performed again after removing outliers. The leave-one-out analysis was conducted within the IVW method to assess the influence of individual variants on the observed association. To identify specific drug targets driving the causal effect, we also examined the causal effect of each drug target within different AHM classes on PD.

### Statistical analysis

The causal effect of SBP, DBP, and PP on outcomes was scaled to a 10-mmHg increment in BP levels. In contrast, associations of AHMs with outcomes were scaled to a 10-mmHg decrease in SBP to represent the therapeutic inhibition of different AHM classes. The association is considered to be significant after Bonferroni correction for BP traits [*p* < 0.016 (0.05/3)] and AHMs [*p* < 0.008 (0.05/6)]. A *p*-value above 0.016/0.008 but below 0.05 was considered suggestive of evidence for a potential association. False-discovery rate was used to correct for multiple testing when calculating the effect of a single drug target within different AHMs on PD, and an adjusted *p*-value of IVW or Wald ratio less than 0.05 is considered to be significant. The main statistical analyses were performed using ‘TwoSampleMR’ (V.0.5.6) in the R package (V.4.1.3) (Hemani et al., 2018).

## Results

### Positive control analyses

**FIGURE 2:**
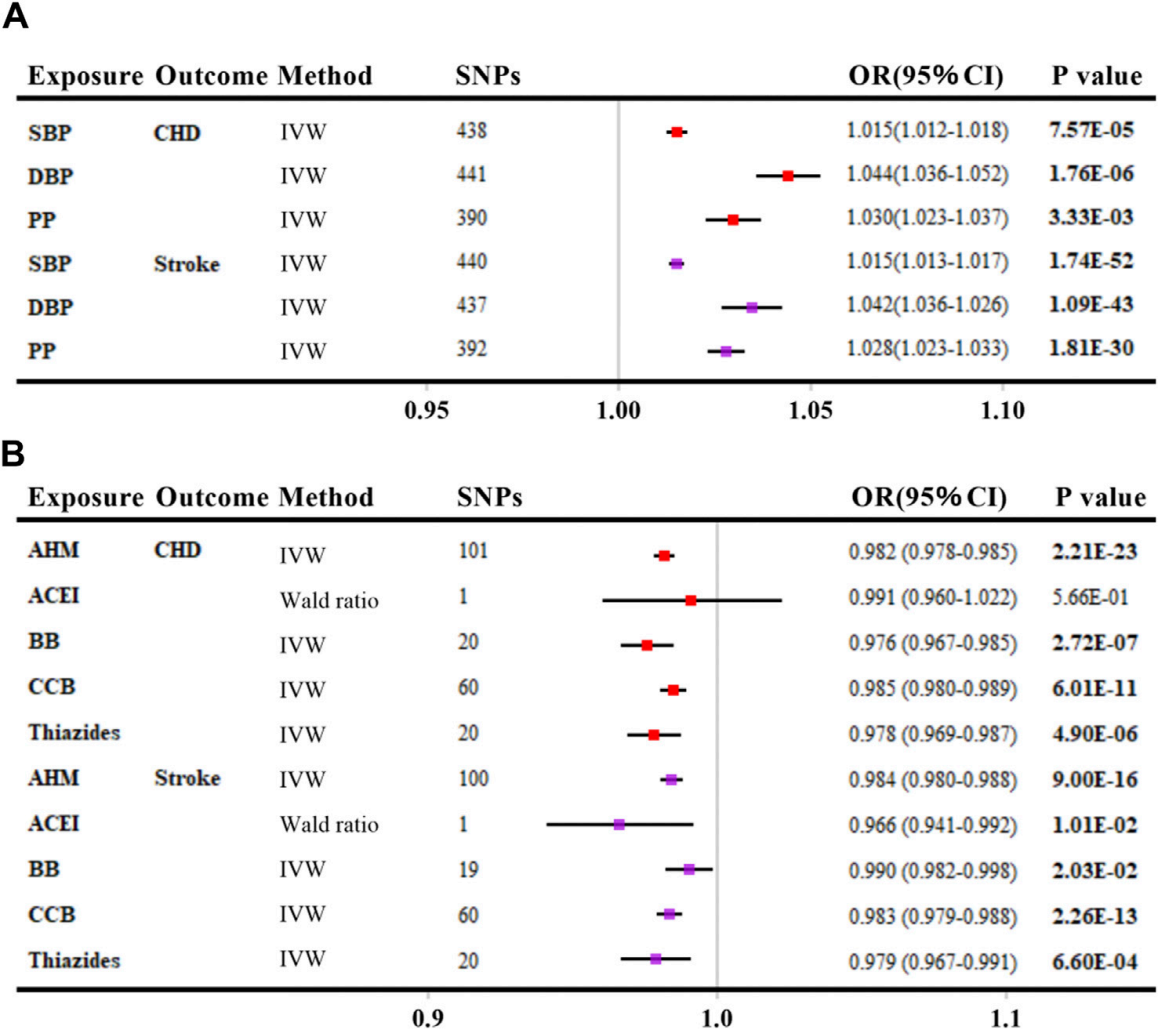
Positive control analyses investigating the effects of blood pressure **(A)** and antihypertensive medications **(B)** on coronary heart disease and stroke. The linkage disequilibrium thresholds of *R* ^*2*^ were set as 0.001 for BP and 0.4 for AHMs. OR and 95% CIs were scaled to each 10-mmHg increment for BP traits and 10-mmHg lower in SBP for AHMs by ‘encoding region-based method’. *p*-value less than 0.05 was depicted in bold. Abbreviations: SBP, systolic blood pressure; DBP, diastolic blood pressure; PP, pulse pressure; CHD, coronary heart disease; IVW, inverse variance weighted; AHM, antihypertensive medications; ACEI, angiotensin-converting enzyme inhibitors; BB, β-blockers; CCB, calcium channel blockers; SNP, single nucleotide polymorphism; OR, odds ratio; CI, confidence interval.

As shown in Figure 2A, genetically elevated SBP, DBP, and PP were all positively associated with the risk of CHD and stroke (per 10-mmHg increment, all *p* values <0.016), consistent with previous evidence (Georgakis et al., 2020; Wan et al., 2021). When looking into AHMs (Figure 2B), overall AHM, BB, CCB, and Thiazides were inversely associated with the risk of CHD (Gill et al., 2019) (per 10-mmHg lower, all *p* values <0.008) except for ACEI. Besides, overall AHM, ACEI, BB, CCB, and Thiazides were associated with a reduced risk of stroke (per 10-mmHg lower, all *p* values <0.05). In summary, positive control analyses confirmed the validity of the predefined IV selection methodology.

### Genetically determined BP with PD risk (main outcome)

**FIGURE 3:**
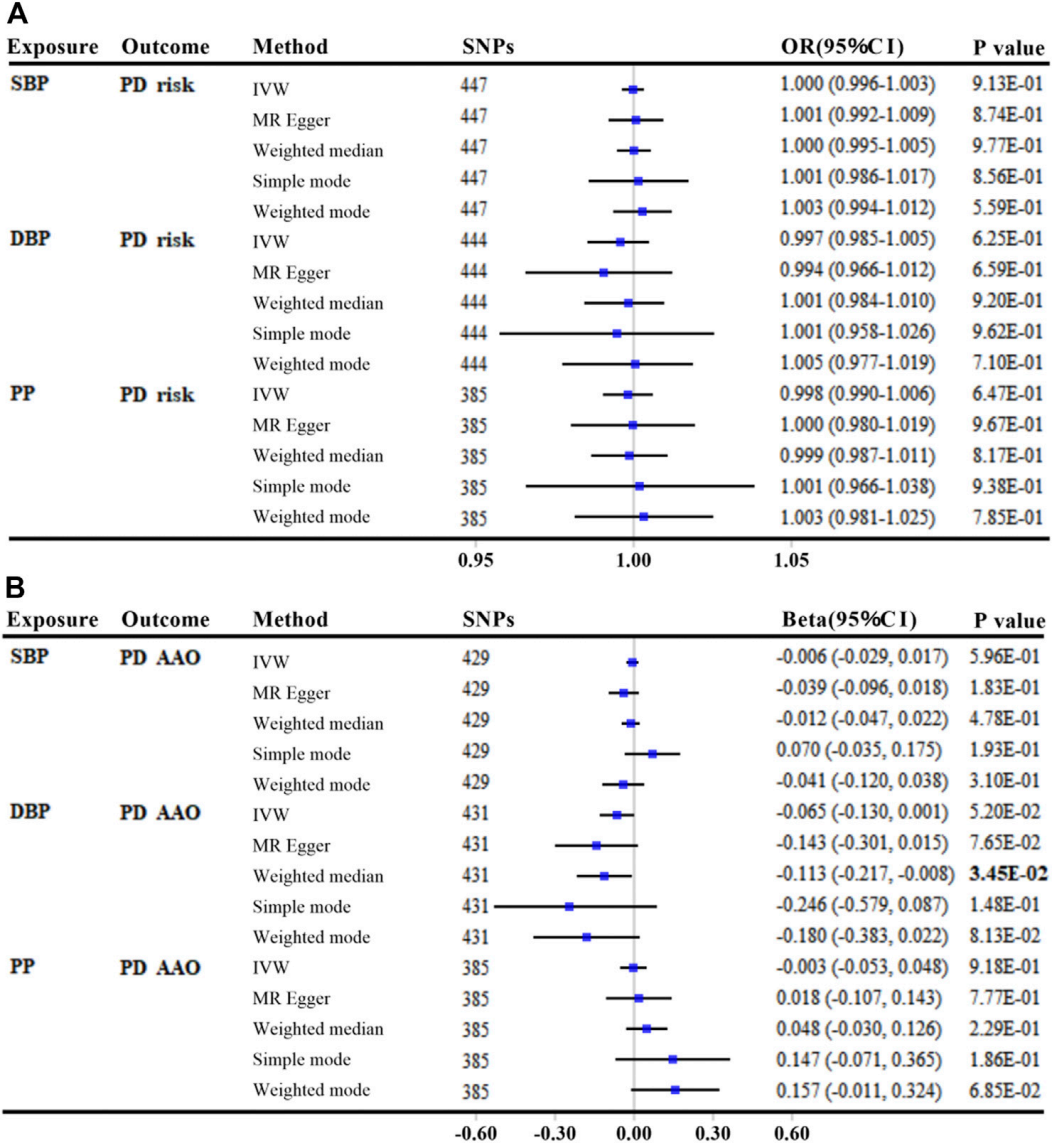
MR analyses between genetically predicted blood pressure and Parkinson’s disease risk **(A)** and age at onset **(B)**. The linkage disequilibrium threshold of *R* ^*2*^ was set as 0.001. OR/Beta & 95% CI was scaled to each 10-mmHg increment in BP traits. *p*-value less than 0.05 was depicted in bold. Abbreviations: MR, Mendelian randomization; SBP, systolic blood pressure; DBP, diastolic blood pressure; PP, pulse pressure; PD, Parkinson’s disease; IVW, inverse variance weighted; AAO, age at onset; SNP, single nucleotide polymorphism; OR, odds ratio; CI, confidence interval.

We found no association of genetically elevated SBP, DBP, or PP with PD risk, with *p* values greater than 0.05 in all MR analyses (per 10-mmHg increment) (Figure 3A and Supplementary Table S4). The effects of SBP and DBP on the risk of PD were also similar using alternative genetic instruments derived from the UK Biobank, which were not adjusted for BMI (Supplementary Table S5).

### Genetically determined BP with PD AAO (secondary outcome)

There was little evidence of an association between either genetically predicted SBP or PP with PD AAO (all *p* values >0.05, per 10-mmHg increment) (Figure 3B and Supplementary Table S6). Using secondary GWAS statistics of SBP and DBP from the UK Biobank, we get similar results that each 10-mmHg increment in DBP was suggestively associated with a younger PD AAO only by MR Egger method (Beta: −3.734; 95% CI: −6.686, −0.783; *p* = 1.38E-02) (Supplementary Table S7).

### Genetically therapeutic inhibition of AHM with PD risk (main outcome)

**FIGURE 4:**
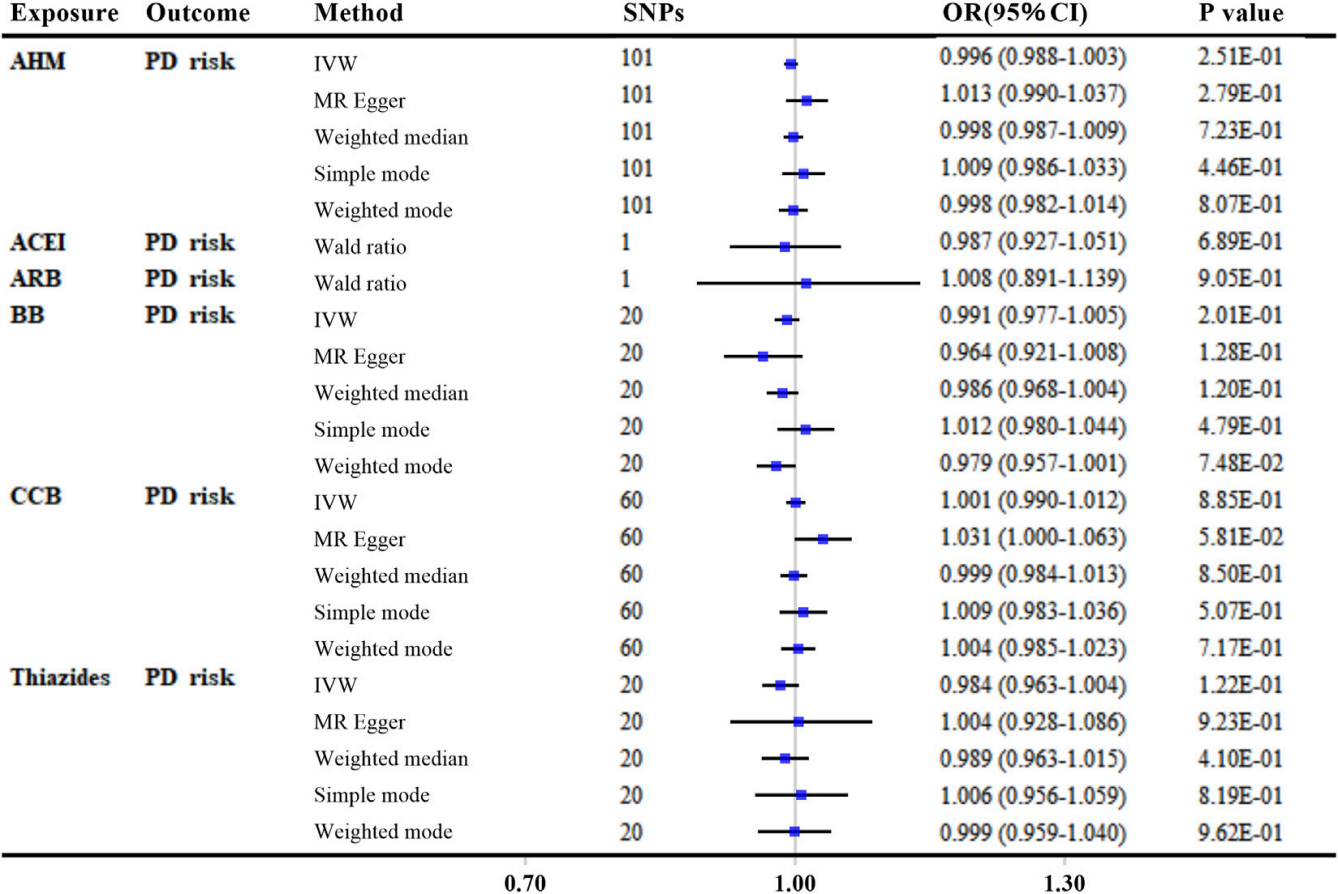
MR analyses of genetically predicted antihypertensive medications with Parkinson’s disease risk by “encoding region-based method”. Genetic proxies for AHMs were selected by “encoding region-based method”. The linkage disequilibrium threshold of *R* ^*2*^ was set as 0.4. OR and 95% CI was scaled to each 10-mmHg lower in SBP. *p*-value less than 0.05 was depicted in bold. Abbreviations: MR, Mendelian randomization; AHM, antihypertensive medications; ACEI, angiotensin-converting enzyme inhibitors; ARB, angiotensin receptor blockers; BB, β-blockers; CCB, calcium channel blockers; PD, Parkinson’s disease; IVW, inverse variance weighted; SNP, single nucleotide polymorphism; OR, odds ratio; CI, confidence interval.

In the main analyses using ‘encoding region-based method’, there was no evidence that reducing SBP affected the risk of PD *via* the protein targets in overall AHM or different AHM classes (Figure 4 and Supplementary Table S8). When more stringent LD thresholds were set at *R* ^*2*^ < 0.1 and *R* ^*2*^ < 0.001, the results showed consistently null associations with the primary analyses for overall AHM and different AHMs (Supplementary Table S9).

In the additional analyses using “eQTL-based method”, neither genetic proxies for overall AHM nor those of different AHMs were associated with PD risk (all *p* values >0.05, Supplementary Figure S1 and Supplementary Table S8). Additionally, MR analyses using more stringent LD thresholds (*R* ^*2*^ < 0.1 and *R* ^*2*^ < 0.001, respectively) remained non-significant for associations of overall AHM and different AHMs with PD risk (all *p* values >0.05, Supplementary Table S10).

Furthermore, individual target analyses only identified *CACNA1H* (OR: 1.431; 95% CI: 1.215–1.686; Adjusted *p* = 5.37E-04; per 10-mmHg lower) by “eQTL-based method” as a significant target that may drive the causal effect of CCB on PD risk (Supplementary Table S11).

### Genetically therapeutic inhibition of AHM with PD AAO (secondary outcome)

In the primary analyses using “encoding region-based method”, although it was suggestive that genetic proxies for BB were associated with a delayed PD AAO (Beta: 0.115; 95% CI: 0.021,0.208; *p* = 1.63E-2; per 10-mmHg lower; *R* ^*2*^ < 0.4) (Supplementary Figure S2 and Supplementary Table S12), more stringent LD thresholds (*R* ^*2*^ < 0.1 and *R* ^*2*^ < 0.001, respectively) failed to support this association in all MR methods (all *p* values >0.05, Supplementary Table S13).

In the secondary analyses using ‘eQTL-based method’, the results were suggestive of an association between thiazides and PD AAO only by IVW method (Beta: −0.287; 95% CI: −0.545, −0.030; *p* = 2.86E-02, Supplementary Figure S3 and Supplementary Table S12). However, restricting LD thresholds to *R* ^*2*^ < 0.1 or *R* ^*2*^ < 0.001 made the results null (Supplementary Table S14).

Analyzing individual targets identified *ADRB1* (Beta: 0.192; 95% CI: 0.070, 0.314; adjusted *p* = 3.19E-02; per 10-mmHg lower) by ‘encoding region-based method’ and *KCNH2* (Beta: −0.650; 95% CI: −0.983, −0.318; adjusted *p* = 3.64E-03; per 10-mmHg lower) by “eQTL-based method” as significant targets that may drive the causal effect of BB on PD AAO (Supplementary Table S15).

### Heterogeneity and horizontal pleiotropy

Although there was limited evidence of heterogeneity and horizontal pleiotropy in MR analyses for BP traits and different AHMs, the MR-PRESSO approach and leave-one-out analyses indicated that our estimates were overall stable (Table 1 and Supplementary Figures S4–S7).

**TABLE 1:** Heterogeneity, horizontal pleiotropy test.

Exposure	Outcome	SNPs	Heterogeneity		Horizontal pleiotropy	MR-PRESSO
			MR Egger	IVW	P	Global test P
SBP	PD risk	447	**4.4E-04**	**4.9E-04**	0.825	6.0E-04^a^
DBP		444	**3.1E-12**	**3.7E-12**	0.793	**< 1.0E-04** ^b^
PP		385	**0.012**	**0.013**	0.875	**0.014** ^a^
SBP	PD AAO	429	**0.002**	**0.002**	0.221	**0.0019** ^a^
DBP		431	0.224	0.222	0.286	0.2136
PP		385	0.107	0.113	0.722	0.1156
AHM	PD risk	101	0.139	0.116	0.123	0.117
ACEI		1	NA	NA	NA	NA
ARB		1	NA	NA	NA	NA
BB		20	0.926	0.895	0.222	0.897
CCB		60	0.088	**0.049**	0.050	0.06
Thiazides		20	0.170	0.199	0.605	0.222
AHM	PD AAO	95	0.403	0.427	0.670	0.417
ACEI		1	NA	NA	NA	NA
BB		19	0.555	0.552	0.327	0.562
CCB		58	0.518	0.555	0.924	0.579
Thiazides		17	0.308	0.343	0.522	0.342
AHM*	PD risk	73	0.215	0.222	0.414	0.233
ACEI*		2	NA	0.593	NA	NA
ARB*		6	0.745	0.808	0.592	0.813
BB*		17	0.432	0.415	0.280	0.392
CCB*		31	**0.041**	0.050	0.687	0.066
Thiazides*		17	0.302	0.367	0.937	0.339
AHM*	PD AAO	71	0.482	0.389	0.054	0.378
ACEI*		2	NA	0.877	NA	NA
ARB*		6	0.490	0.537	0.462	0.578
BB*		17	0.156	**0.034**	**0.035**	**0.034** ^b^
CCB*		30	0.901	0.912	0.513	0.910
Thiazides*		16	0.460	0.484	0.418	0.546

*p*-value less than 0.05 was depicted in bold. * Variants selected by ‘eQTL-based method’ for AHMs.

^a^ No outliers identified with the MR-PRESSO, approach.

^b^ *p*-value remained non-significant after removing outliers, including rs4954192 (*ACMSD*), rs72842207(*BAG3*), rs79724577 (*ARHGAP27*), and rs80095680(*BCL7C*) for PP, and including rs2484294 (-) for BB.

Abbreviation: SNP, single nucleotide polymorphism; MR, mendelian randomization; IVW, inverse-variance weighted; MR-PRESSO, MR, pleiotropy residual sum and outlier; SBP, systolic blood pressure; DBP, diastolic blood pressure; PP, pulse pressure; AHM, antihypertensive medications, BB, β-blockers; CCB, calcium channel blockers; PD, Parkinson’s disease; AAO, age of onset; NA, not available.

## Discussion

To the best of our knowledge, the current study firstly assessed the causal effects of BP and antihypertensive medications on PD using two-sample MR analyses. However, our results failed to support that BP was causally associated with PD risk and AAO. There was also limited evidence that lowering SBP *via* the targets of first-line antihypertensive drugs affected PD. In summary, our study could help validate the role of BP in the pathogenesis of PD and avoid overestimating the repurposing role of antihypertensive drugs in PD prevention.

Whether hypertension represents a risk factor for PD has not been fully elucidated for a long time, and observational studies in the literature that examined the risk for PD yielded conflicting results. Two studies reported that the risk of PD was not significantly related to high BP(Simon et al., 2007; Tan et al., 2008). In comparison, two other studies suggested that high BP slightly increased the risk of PD (Qiu et al., 2011; Lai et al., 2014), at least in women with arterial hypertension (Qiu et al., 2011). In comparison, another study instead reported that high BP exerted a protective role against the development of PD (Paganini-Hill, 2001). However, all these studies differed in sample size, geographic origin, and duration of the follow-up of the cohort population. Furthermore, concerning the role of antihypertensive drugs in the risk of developing PD, studies showed that among different classes of AHMs, dihydropyridine CCB, but not non-dihydropyridine CCB, may be associated with a reduced risk of PD (Tseng et al., 2021). For β-Adrenoceptor acting agents, a recent meta-analysis of epidemiologic studies suggests that the intake of β-adrenoceptor antagonists, including propranolol and metoprolol, may serve as a risk factor for PD development (Saengphatrachai et al., 2021).

However, PD may have a long prodromal stage up to decades before PD diagnosis, characterized by non-motor dysfunction such as SBP drop, sleep disturbances, and constipation (Postuma et al., 2013; Chen and Ritz, 2018). The prodromal population may be more susceptible to the diagnosis of abnormal BP and an altered tendency to use AHMs, resulting in the inability to accurately define the history of hypertension and AHMs before PD diagnosis in previous cohort studies. Apart from reverse causation, earlier epidemiologic studies could have been subject to unmeasured confounders, such as socioeconomic status, mood disorders, physical activity, and other drug use.

Due to the limitations of observational studies, MR analyses provide an attractive prospect to identify risk or protective factors. Although we provide robust results for the null association of BP and AHMs with PD using primary and secondary MR analyses, we should interpret these results cautiously. In this study, we assumed that the estimated effect of the targets of overall AHM or different AHMs on PD risk or AAO acted through SBP-lowering, but there is potentially an alternative mechanism by which the targets can affect PD. Hence, our null results for overall AHM and common AHM classes do not rule out possible benefits *via* competing mechanisms by using this drug for PD prevention. Future larger GWAS will be needed to verify that our results did not generate by accident. Furthermore, more precise mapping and mechanistic studies of targets for AHMs will also help elucidate the pathogenesis of PD.

The present study has some strengths. First, positive control analyses were performed to validate the IV selection strategies and confirmed that the approach was appropriate. Additionally, genetic proxies for AHMs were hypothesized to act through the SBP-lowering effect, which would help us understand the antihypertensive role in PD etiology. Thirdly, we included pharmacologically active targets with known biological functions to proxy each AHM class, contributing to better homogeneity of selected IVs. Last, we included AAO as a secondary outcome for PD risk, further supporting the findings from the main outcome.

This study has several limitations. First, MR analysis assumes that the SNPs selected as IVs for BP traits and AHMs influence PD only through the exposure of interest (no pleiotropic effects). Although there did exist some pleiotropic effects for genetic proxies of BP traits, BB, and CCB in our study, multiple methods confirmed the stability of our results. Second, considering the limited genetic proxies identified, the power of MR analyses for ACEI and ARB may be limited. Third, since all of the participants are mainly of European ancestry, the results of this study are not necessarily valid for other ethnic groups.

In conclusion, this study found little evidence that BP and antihypertensive drugs would affect PD risk and AAO. Future studies should consider our research, combined with other sources of evidence, to obtain a reliable answer about the role of hypertension and the potential repurposing of antihypertensives for PD prevention.

## Data Availability

The original contributions presented in the study are included in the article/Supplementary Material, further inquiries can be directed to the corresponding authors.
