# Supplementary material for: Protective effect of antihypertensive drugs on the risk of Parkinson’s disease lacks causal evidence from mendelian randomization
Source: Front Pharmacol. 2023 Feb 23;14:1107248. doi: 10.3389/fphar.2023.1107248 (PMC9995445; doi:10.3389/fphar.2023.1107248)
Supplement: Supplementary file 1 [file Presentation1.pdf]

Exposure	Outcome	Method	SNPs	OR(95% CI)	P value
**AHM**	**PD risk**	IVW	73	0.999 (0.986-1.013)	8.76E-01
		MR Egger	73	0.987 (0.957-1.019)	4.22E-01
		Weighted median	73	0.997 (0.978-1.017)	7.88E-01
		Simple mode	73	1.005 (0.966-1.047)	7.91E-01
		Weighted mode	73	0.997 (0.975-1.021)	8.32E-01
**ACEI**	**PD risk**	IVW	2	0.979 (0.926-1.035)	4.57E-01
**ARB**	**PD risk**	Inverse variance weighted	6	1.005 (0.937-1.078)	8.87E-01
		MR Egger	6	0.782 (0.334-1.830)	6.01E-01
		Weighted median	6	1.017 (0.931-1.110)	7.09E-01
		Simple mode	6	1.034 (0.915-1.168)	6.17E-01
		Weighted mode	6	1.035 (0.912-1.175)	6.15E-01
**BB**	**PD risk**	IVW	17	0.997 (0.974-1.021)	7.97E-01
		MR Egger	17	0.970 (0.919-1.023)	2.79E-01
		Weighted median	17	0.978 (0.947-1.010)	1.72E-01
		Simple mode	17	1.044 (0.981-1.111)	1.91E-01
		Weighted mode	17	0.981 (0.948-1.015)	2.84E-01
**CCB**	**PD risk**	IVW	31	1.002 (0.980-1.025)	8.48E-01
		MR Egger	31	0.993 (0.945-1.043)	7.85E-01
		Weighted median	31	1.006 (0.981-1.033)	6.26E-01
		Simple mode	31	1.001 (0.964-1.040)	9.44E-01
		Weighted mode	31	1.005 (0.977-1.034)	7.23E-01
**Thiazides**	**PD risk**	IVW	17	0.998 (0.962-1.036)	9.24E-01
		MR Egger	17	0.993 (0.874-1.128)	9.18E-01
		Weighted median	17	1.014 (0.960-1.071)	6.21E-01
		Simple mode	17	1.040 (0.945-1.145)	4.30E-01
		Weighted mode	17	1.031 (0.941-1.130)	5.17E-01

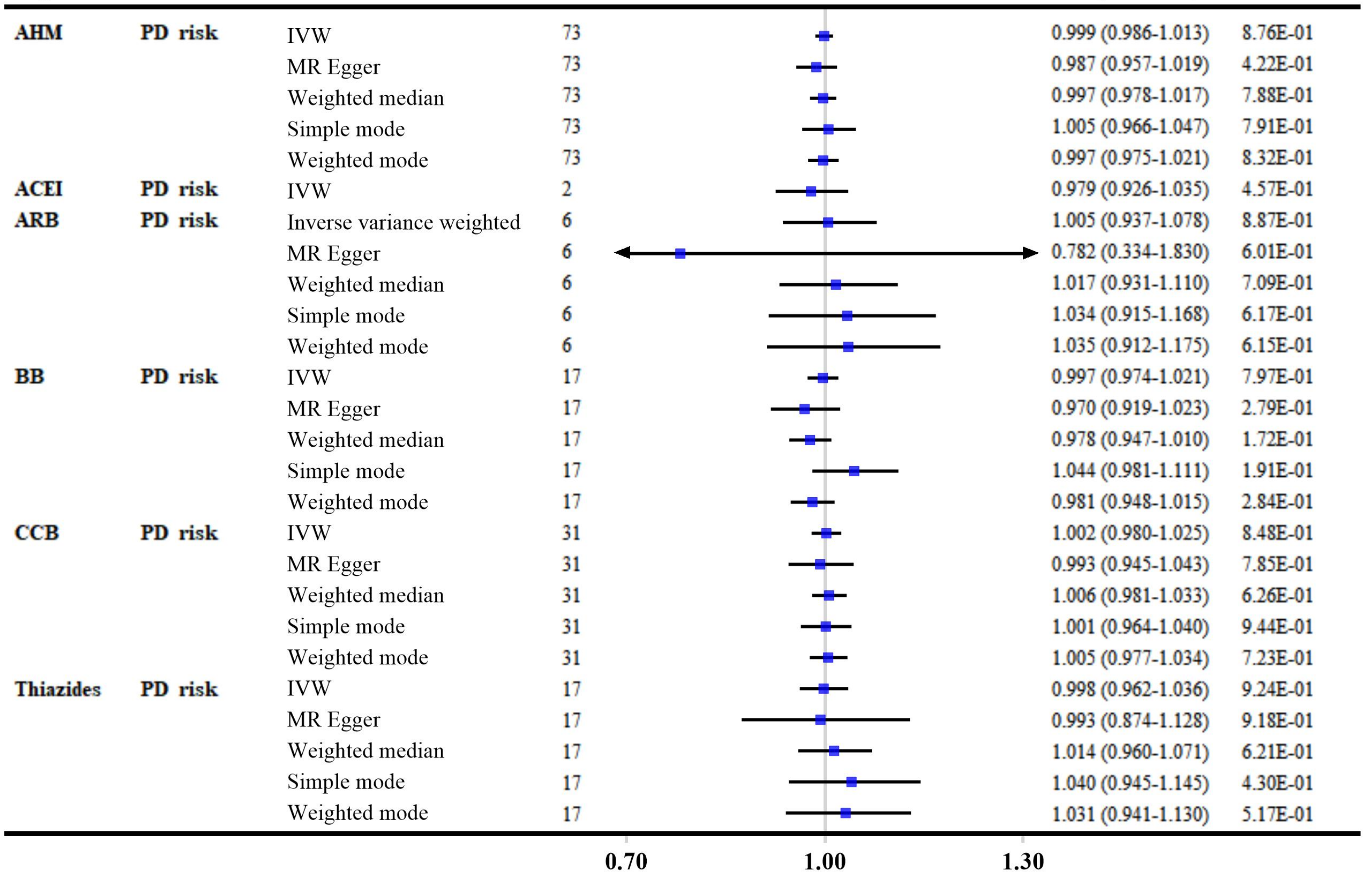

Figure S1. MR analyses of genetically predicted antihypertensive medications with Parkinson's disease risk by 'eQTL-based method'.

Genetic proxies for AHMs were selected by 'eQTL-based method'. The linkage disequilibrium threshold of R^2 was set as 0.4. OR & 95% CI was scaled to each 10-mmHg lower in SBP. *P* value less than 0.05 was depicted in bold.

Abbreviations: AHM, antihypertensive medications; ACEI, angiotensin-converting enzyme inhibitors; ARB, angiotensin receptor blockers; BB, β-blockers; CCB, calcium channel blockers; PD, Parkinson's disease; IVW, inverse variance weighted; SNP, single nucleotide polymorphism; OR, odds ratio; CI, confidence interval.

Exposure	Outcome	Method	SNPs	Beta(95%CI)	P value
**AHM**	**PD AAO**	IVW	95	0.016 (-0.032, 0.065)	5.03E-01
		MR Egger	95	0.047 (-0.099, 0.193)	5.33E-01
		Weighted median	95	0.028 (-0.047, 0.103)	4.70E-01
		Simple mode	95	0.172 (-0.037, 0.381)	1.10E-01
		Weighted mode	95	-0.073 (-0.238, 0.092)	3.86E-01
**ACEI**	**PD AAO**	Wald ratio	1	0.168 (-0.246, 0.583)	4.26E-01
**BB**	**PD AAO**	IVW	19	0.115 (0.021, 0.208)	**1.63E-02**
		MR Egger	19	0.260 (-0.038, 0.558)	1.05E-01
		Weighted median	19	0.180 (0.050, 0.310)	**6.71E-03**
		Simple mode	19	0.230 (-0.015, 0.475)	8.29E-02
		Weighted mode	19	0.227 (0.048, 0.406)	**2.27E-02**
**CCB**	**PD AAO**	IVW	58	-0.028 (-0.090, 0.034)	3.75E-01
		MR Egger	58	-0.036 (-0.219, 0.146)	6.97E-01
		Weighted median	58	-0.065 (-0.163, 0.033)	1.93E-01
		Simple mode	58	0.034 (-0.163, 0.231)	7.38E-01
		Weighted mode	58	-0.068 (-0.208, 0.072)	3.46E-01
**Thiazides**	**PD AAO**	IVW	17	0.008 (-0.133, 0.148)	9.15E-01
		MR Egger	17	-0.16 (-0.681, 0.361)	5.56E-01
		Weighted median	17	0.114 (-0.075, 0.303)	2.37E-01
		Simple mode	17	0.194 (-0.195, 0.583)	3.43E-01
		Weighted mode	17	0.194 (-0.137, 0.526)	2.68E-01

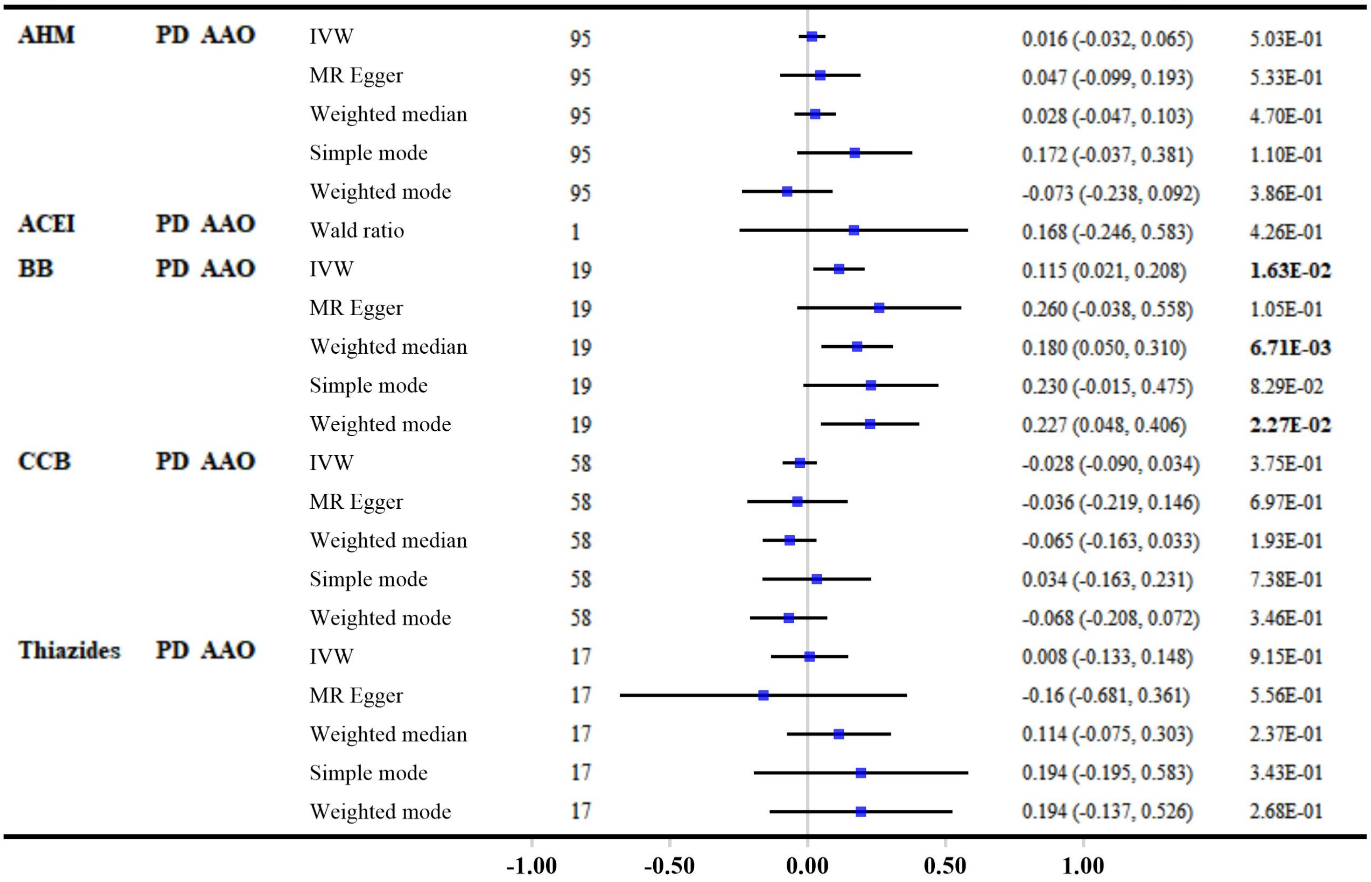

Figure S2. MR analyses of genetically predicted antihypertensive medications with Parkinson's disease age at onset by 'encoding region-based method'.

Genetic proxies for AHMs were selected by 'encoding region-based method'. The linkage disequilibrium threshold of R^2 was set as 0.4. Beta & 95% CI was scaled to each 10-mmHg lower in SBP. *P* value less than 0.05 was depicted in bold.

Abbreviations: AHM, antihypertensive medications; ACEI, angiotensin-converting enzyme inhibitors; BB, β-blockers; CCB, calcium channel blockers; PD, Parkinson's disease; IVW, inverse variance weighted; AAO, age at onset; SNP, single nucleotide polymorphism; CI, confidence interval.

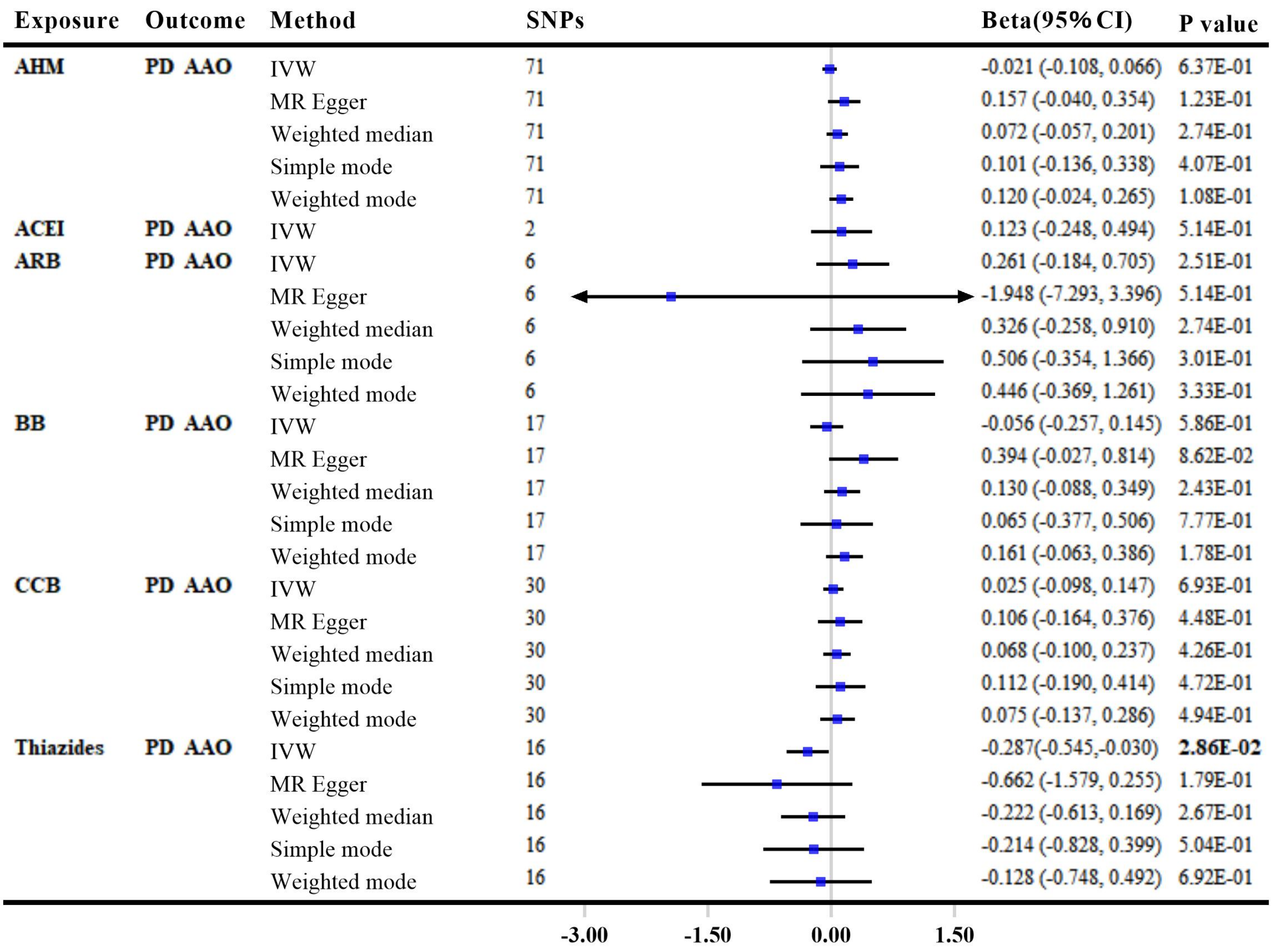

Figure S3. MR analyses of genetically predicted antihypertensive medications with Parkinson's disease age at onset by 'eQTL-based method'.

Genetic proxies for AHMs were selected by 'eQTL-based method'. The linkage disequilibrium threshold of R^2 was set as 0.4. Beta & 95% CI was scaled to each 10-mmHg lower in SBP. *P* value less than 0.05 was depicted in bold.

Abbreviations: AHM, antihypertensive medications; ACEI, angiotensin-converting enzyme inhibitors; ARB, angiotensin receptor blockers; BB, β-blockers; CCB, calcium channel blockers; PD, Parkinson's disease; IVW, inverse variance weighted; AAO, age at onset; SNP, single nucleotide polymorphism; CI, confidence interval.

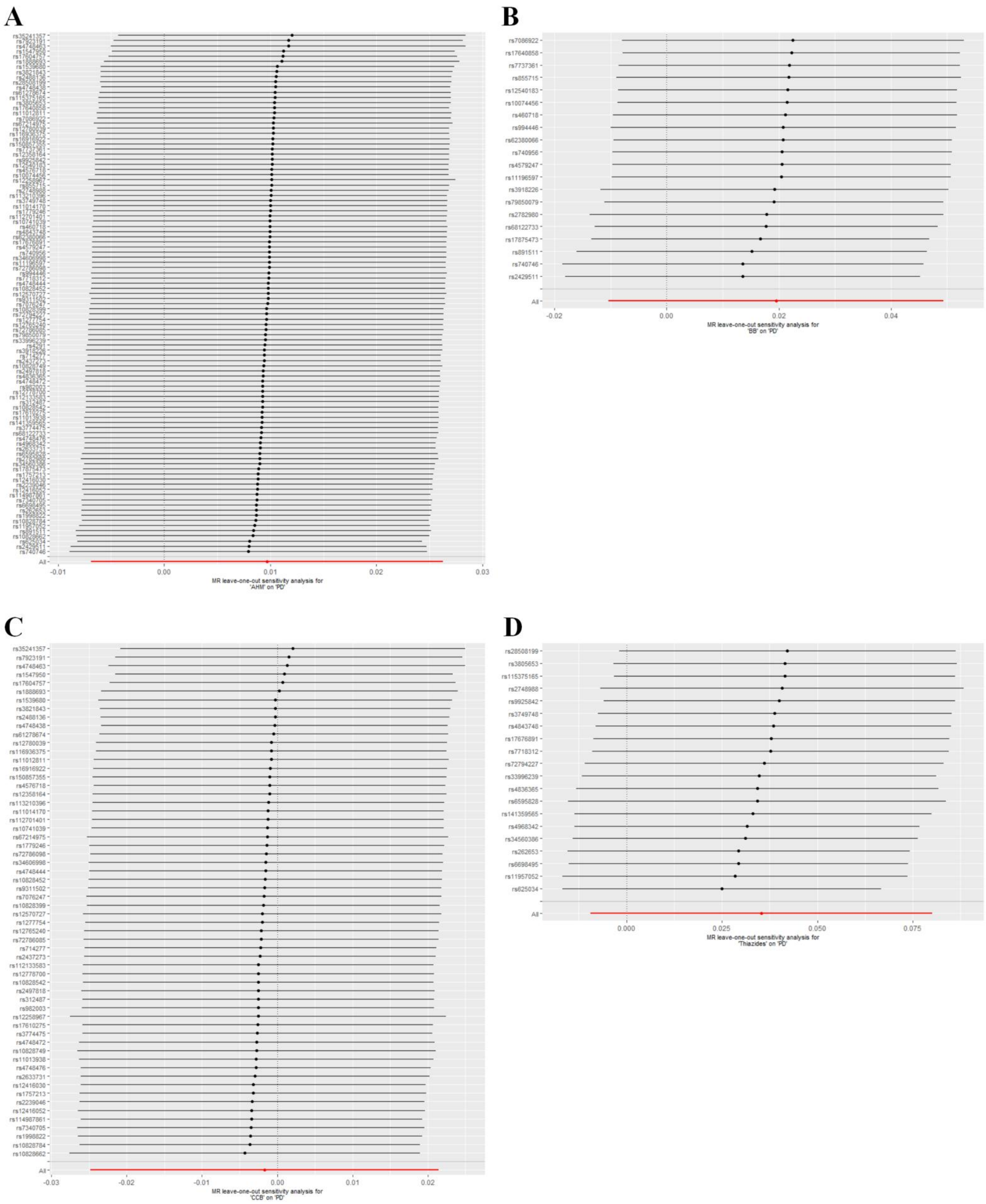

Figure S4. Leave-one-out analyses of MR associations between genetic proxies for overall antihypertensive medications (A), beta-blockers (B), calcium channel blockers (C), and thiazides (D) and Parkinson's disease risk by 'encoding region-based method'.

Abbreviations: AHM, antihypertensive medications; BB, β-blockers; CCB, calcium channel blockers; PD, Parkinson's disease.

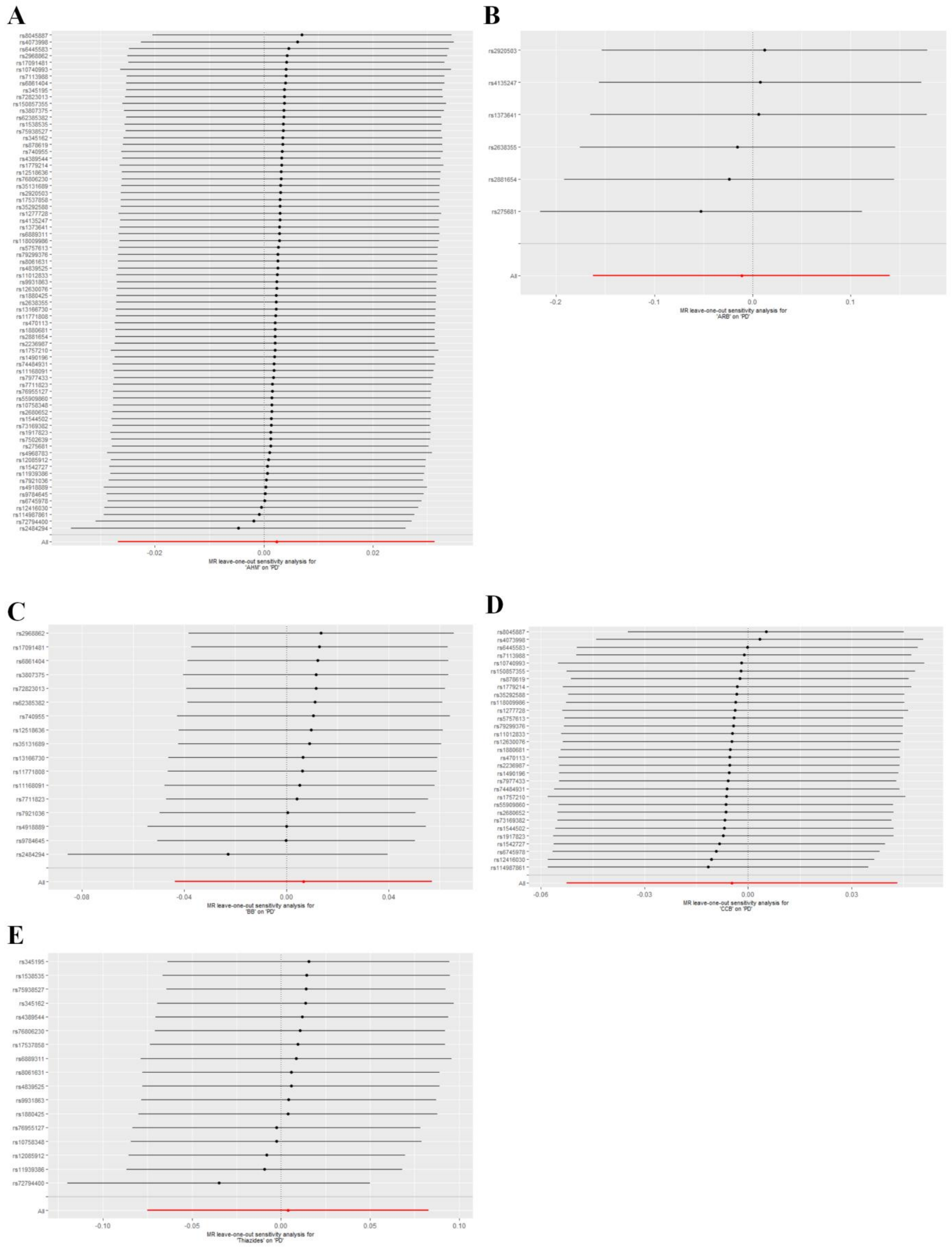

Figure S5. Leave-one-out analyses of MR associations between genetic proxies for overall antihypertensive medications (A), angiotensin receptor blockers (B), beta-blockers (C), calcium channel blockers (D), and thiazides (E) and Parkinson's disease risk by 'eQTL-based method'.

Abbreviations: AHM, antihypertensive medications; ARB, angiotensin receptor blockers; BB, β-blockers; CCB, calcium channel blockers.

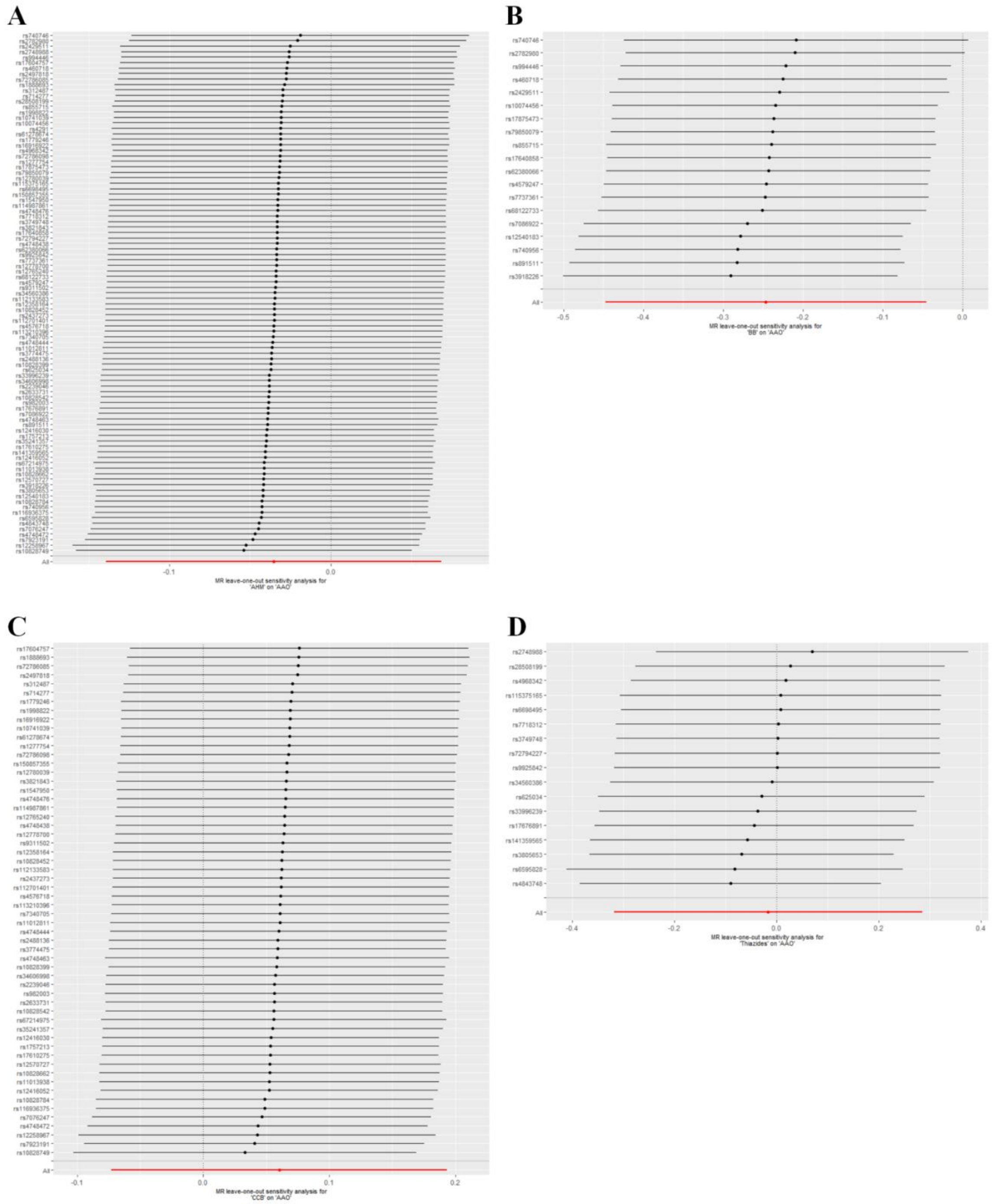

Figure S6. Leave-one-out analyses of MR associations between genetic proxies for overall antihypertensive medications (A), beta-blockers (B), calcium channel blockers (C), and thiazides (D) and Parkinson's disease age at onset by 'encoding region-based method'.

The leave-one-out analysis indicated that rs740746 or rs2782980 significantly drove the potential effect of BB on PD AAO. Removing these two SNPs made the association non-significant (Beta: 0.097; 95% CI: -0.004, 0.197; *P* = 5.92E-02; per 10-mmHg lower; $R^2 < 0.4$).

Abbreviations: AHM, antihypertensive medications; BB, β-blockers; CCB, calcium channel blockers; AAO, age at onset.

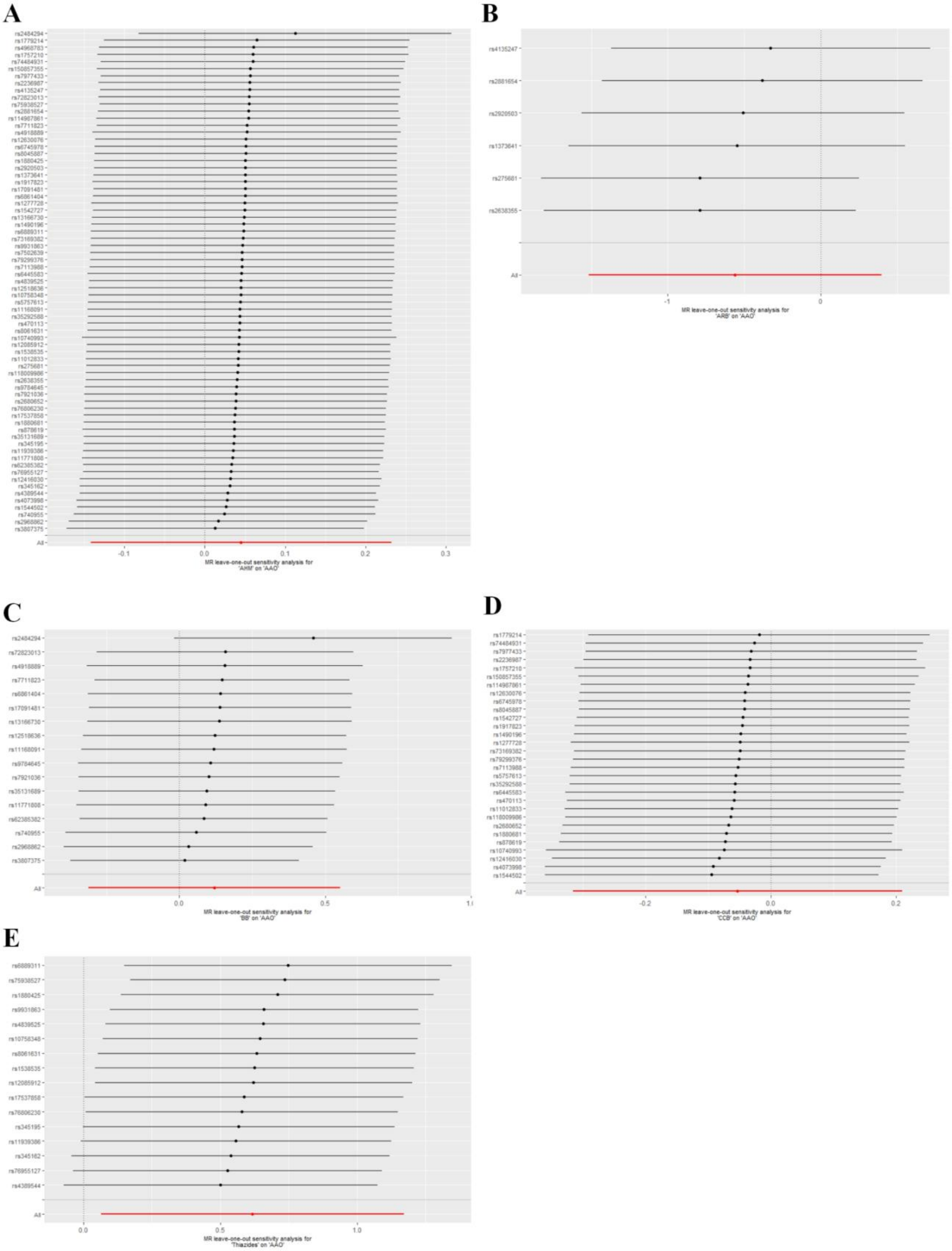

Figure S7. Leave-one-out analyses of MR associations between genetic proxies for overall antihypertensive medications (A), angiotensin receptor blockers (B), beta-blockers (C), calcium channel blockers (D), and thiazides (E) and Parkinson's disease age at onset by 'eQTL-based method'.

The leave-one-out analysis indicated that the weak effect of thiazides on PD AAO depended on rs345162, rs345195, rs11939386, rs76955127, or rs4389544.

Abbreviations: AHM, antihypertensive medications; ARB, angiotensin receptor blockers; BB, β-blockers; CCB, calcium channel blockers; AAO, age at onset.
